# Supplementary figures and images for: Comparing DNA replication programs reveals large timing shifts at centromeres of endocycling cells in maize roots
Source: PLoS Genet. 2020 Oct 14;16(10):e1008623. doi: 10.1371/journal.pgen.1008623 (PMC7588055; doi:10.1371/journal.pgen.1008623)

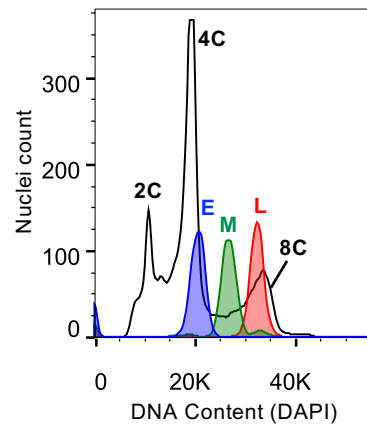

Supplement: S1 Fig — Maize root tip nuclei were isolated from the 1–3 mm root region and sorted on a BD InFlux flow sorter. A small sample from each of the three S-phase sort gates was re-analyzed to determine the purity of the sorted nuclei. Histograms of relative DNA content (DAPI fluorescence) from re-analyzed sorted nuclei are overlaid for early (E), mid (M), and late (L) S-phase gates from the endocycle arc to show the separation between sorted samples. Similar separation was found for sorted early, mid and late nuclei from the mitotic cycle (see S1 Fig in [19]). The histogram of relative DNA content for the entire unsorted nuclei population (black line) is shown for reference. (PDF) [file pgen.1008623.s002.pdf]

S2 Fig.

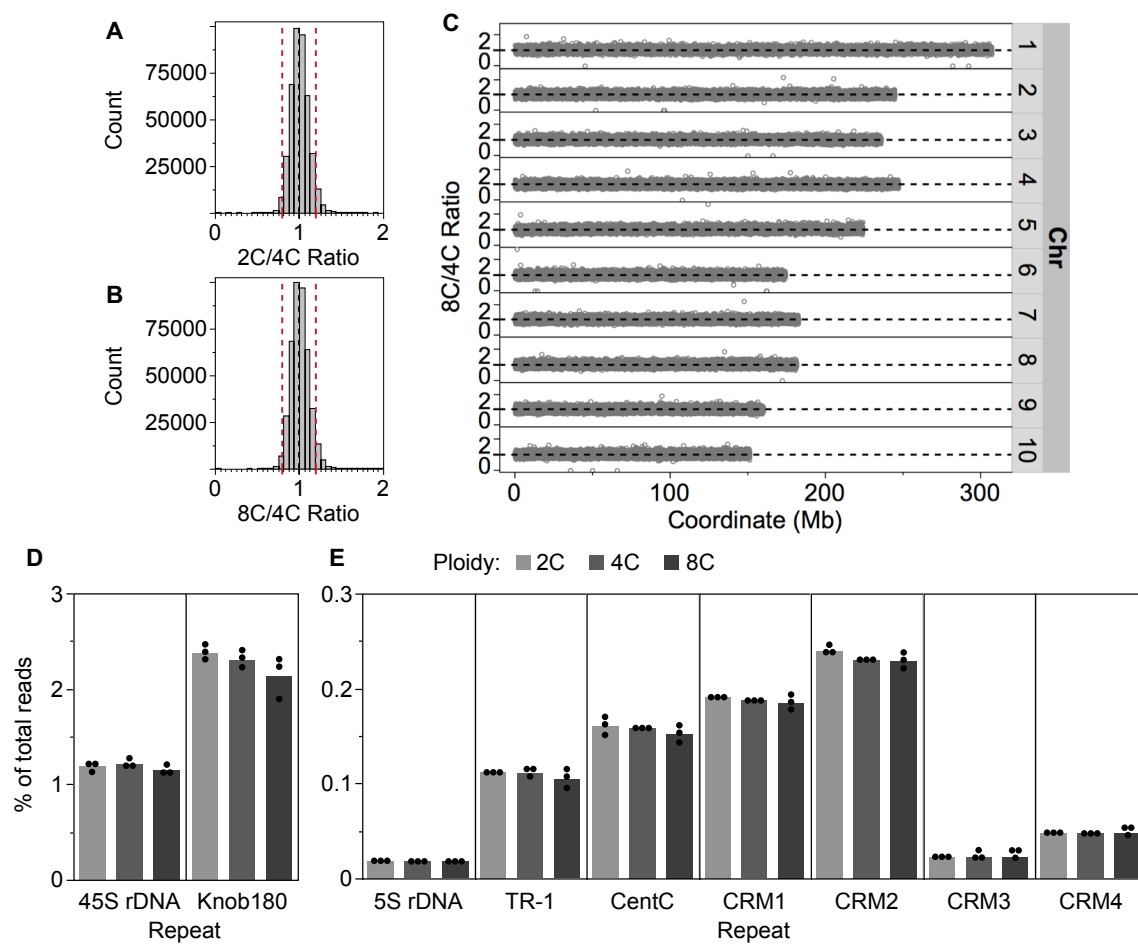

Supplement: S2 Fig — Whole genome sequence data from sorted non S-phase 2C, 4C and 8C nuclei were used to assess copy number per DNA content across the genome. To better represent the copy number of repeat regions, the primary alignment location for each read pair–even those that map to multiple locations–were included in the analysis. (A and B) Histograms of the normalized read frequency ratios, calculated in 5-kb static windows, for 2C/4C (A) and 8C/4C (B) nuclei. The black dashed lines indicate the overall mean and the red dashed lines indicate ± 2 S. D. from the mean. (C) The 8C/4C read frequency ratios plotted as a function of genomic location, which shows that the values outside ± 2 S. D. all occur as singleton 5-kb windows. (D and E) We used consensus sequences for 45S rDNA and knob180 (D), and for 5S rDNA, TR-1, CentC and CRM1–4 families (E) to individually query all of the trimmed whole genome sequence reads using BLAST software and a non-stringent E value to allow for variants of each repeat (S1 Text). The mean percentage of total reads that align to each repeat type was calculated for three biological replicates of 2C, 4C and 8C data. Black dots represent the individual biological replicate values. The apparent slight under-replication of several elements (e.g. knob180 and CRM2) is not statistically significant. (PDF) [file pgen.1008623.s003.pdf]

S3 Fig.

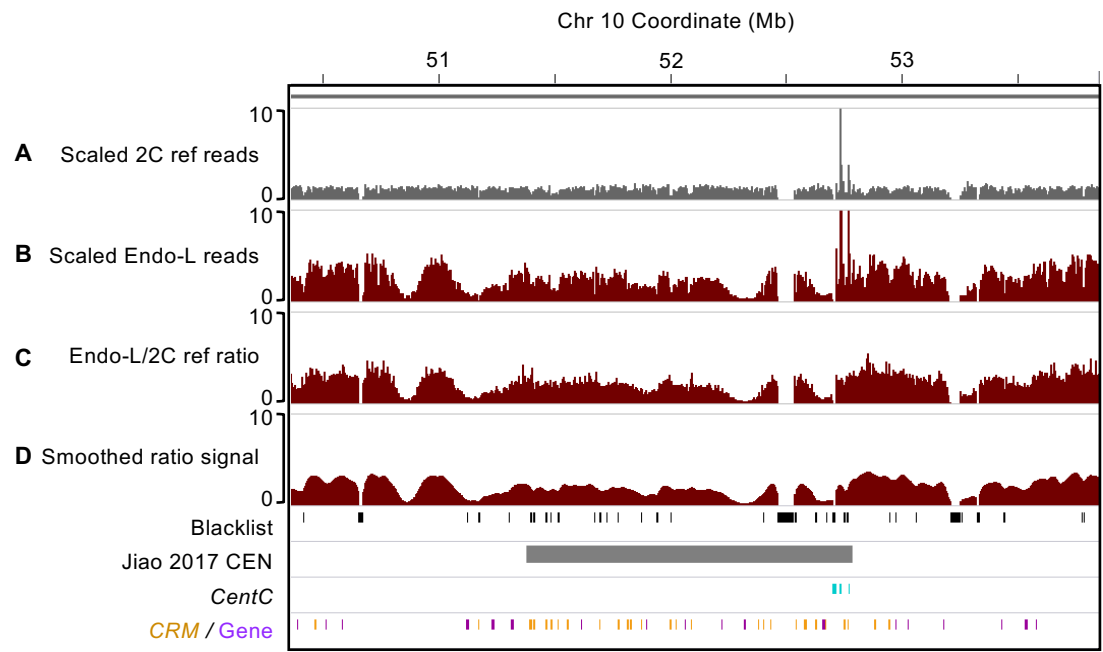

Supplement: S3 Fig — An example region from CEN 10 is shown to illustrate that the pre-replicative 2C reference data effectively normalizes spikes of signal in the S-phase data. (A and B) Read densities were calculated in 3-kb windows for the 2C reference (A) and each S-phase sample (endocycle late profile shown; B). After excluding blacklist regions (e.g. unmappable and multi-mapping regions), reads were scaled for overall sequence depth in each sample. (C) Scaled reads in each S-phase sample were normalized by making a ratio to 2C reference scaled reads in each 3-kb window. (D) Replication signal profiles were smoothed using a Haar wavelet transform to remove noise without altering peak boundaries. (PDF) [file pgen.1008623.s004.pdf]

S4 Fig.

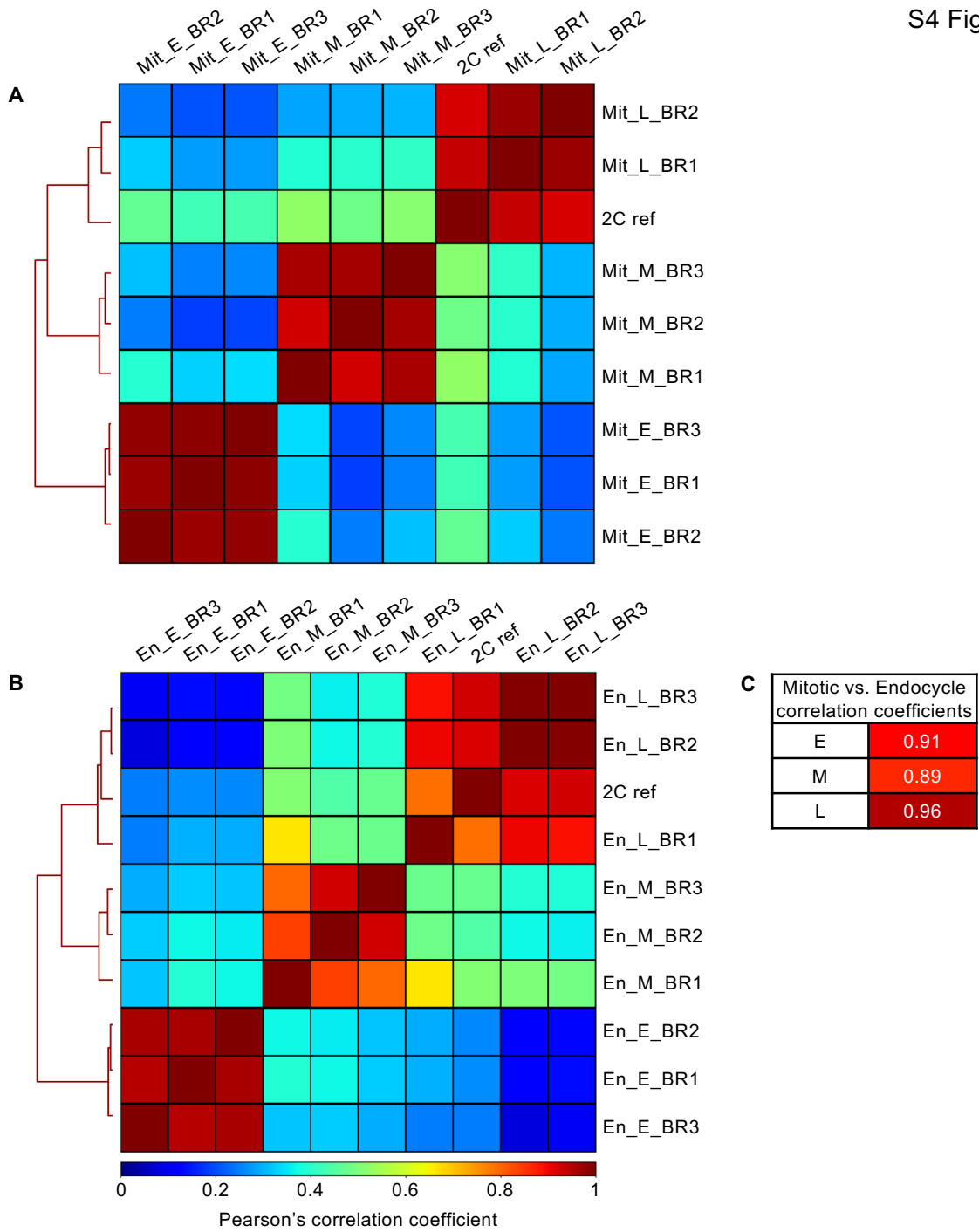

Supplement: S4 Fig — (A and B) Biological replicates (BR) of early (E), mid (M) and late (L) Repli-seq data for the mitotic cycle (Mit; panel A) and endocycle (En; panel B) was analyzed independently using Repliscan [24]. The agreement between biological replicates was assessed by calculating Pearson’s correlation coefficients. (C) The Pearson’s correlation coefficients for E, M, L data between mitotic cycle and endocycle. (PDF) [file pgen.1008623.s005.pdf]

S5 Fig.

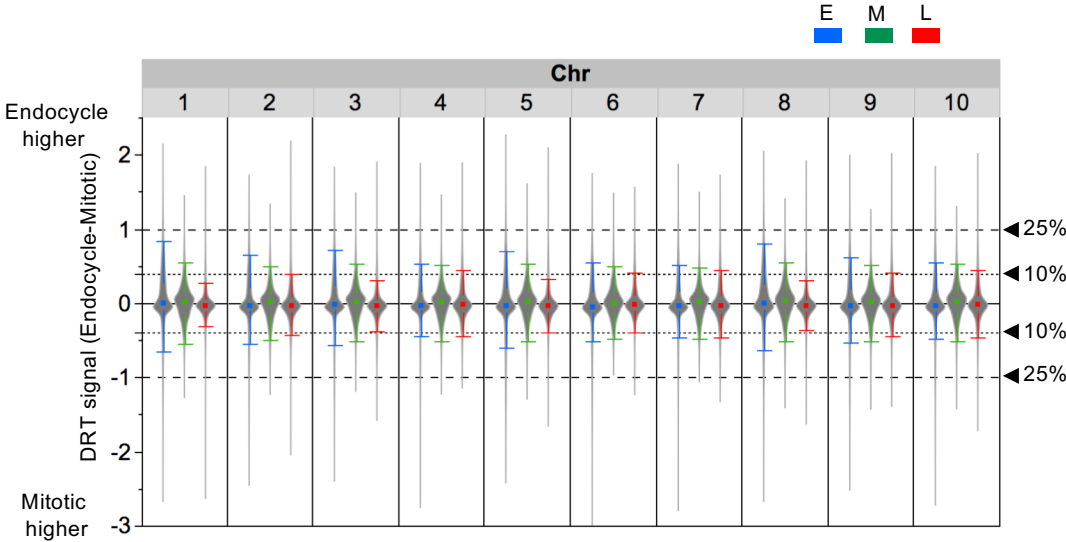

Supplement: S5 Fig — Differences in replication timing (DRT) signal were calculated by subtracting the mitotic signal from the endocycle signal for early (E), mid (M) and late (L) S-phase fractions in each 3-kb window across the genome. The distributions of DRT signal values are represented as violin plots for each chromosome. Median values are indicated by colored squares and 1.5 x IQR of the distribution is indicated by colored whisker lines. Dashed lines indicate the thresholds used in subsequent steps for identifying RATs (≥ 10% and ≥ 25% of the total difference range; S1 Table). (PDF) [file pgen.1008623.s006.pdf]

S6 Fig.

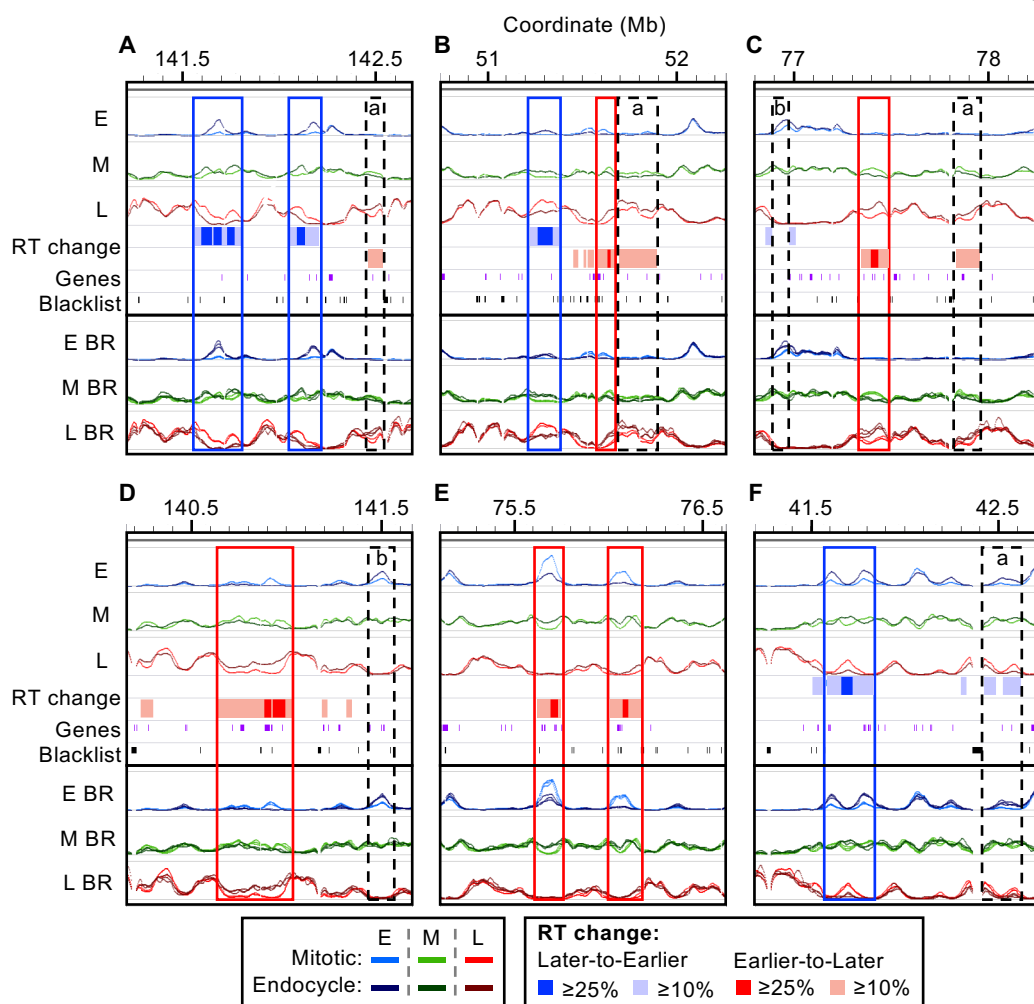

Supplement: S6 Fig — (A–F) Example regions on chromosomes 1 (A), 3 (B), 4 (C), 5 (D), 6 (E) and 7 (F) that include RATs. See main text Fig 2 legend for description. Dashed boxes denote regions with some level of DRT in which the magnitude of the difference did not meet our ≥ 25% criterion (boxes labeled “a” in panels A, B, C and F), or in which the change in one S-phase fraction was not compensated by an opposite change in at least one other S-phase fraction (boxes labeled “b” in panels C and D). (PDF) [file pgen.1008623.s007.pdf]

S8 Fig.

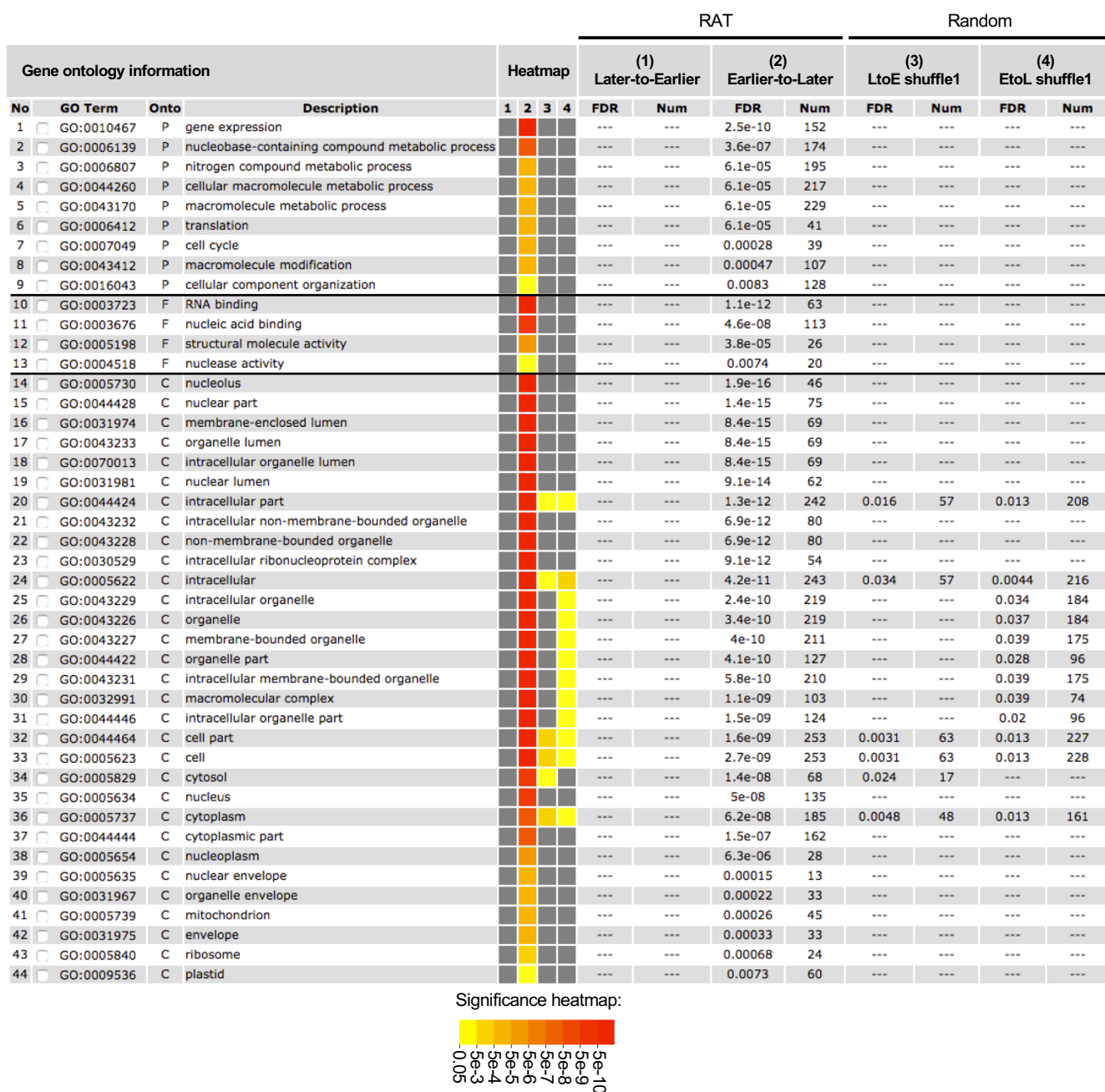

Supplement: S8 Fig — Using the Plant GO slim ontology subset, we identified 44 significant GO terms in the biological process (P), molecular function (F), and cellular component (C) GO categories that were enriched in expressed genes (S1 Text; S3 Spreadsheet) in Earlier-to-Later RATs. Genes in the corresponding randomly shuffled set shared a few of the significantly enriched cellular component terms as genes in Earlier-to-Later RATs, suggesting that these terms may be related to common components of the root, and not RATs specifically. The total number of expressed genes in each input gene list was as follows: Later-to-Earlier RATs, 52; LtoE shuffle1 random regions, 68; Earlier-to-Later RATs, 292; EtoL shuffle1 random regions, 275. (PDF) [file pgen.1008623.s009.pdf]

S9 Fig.

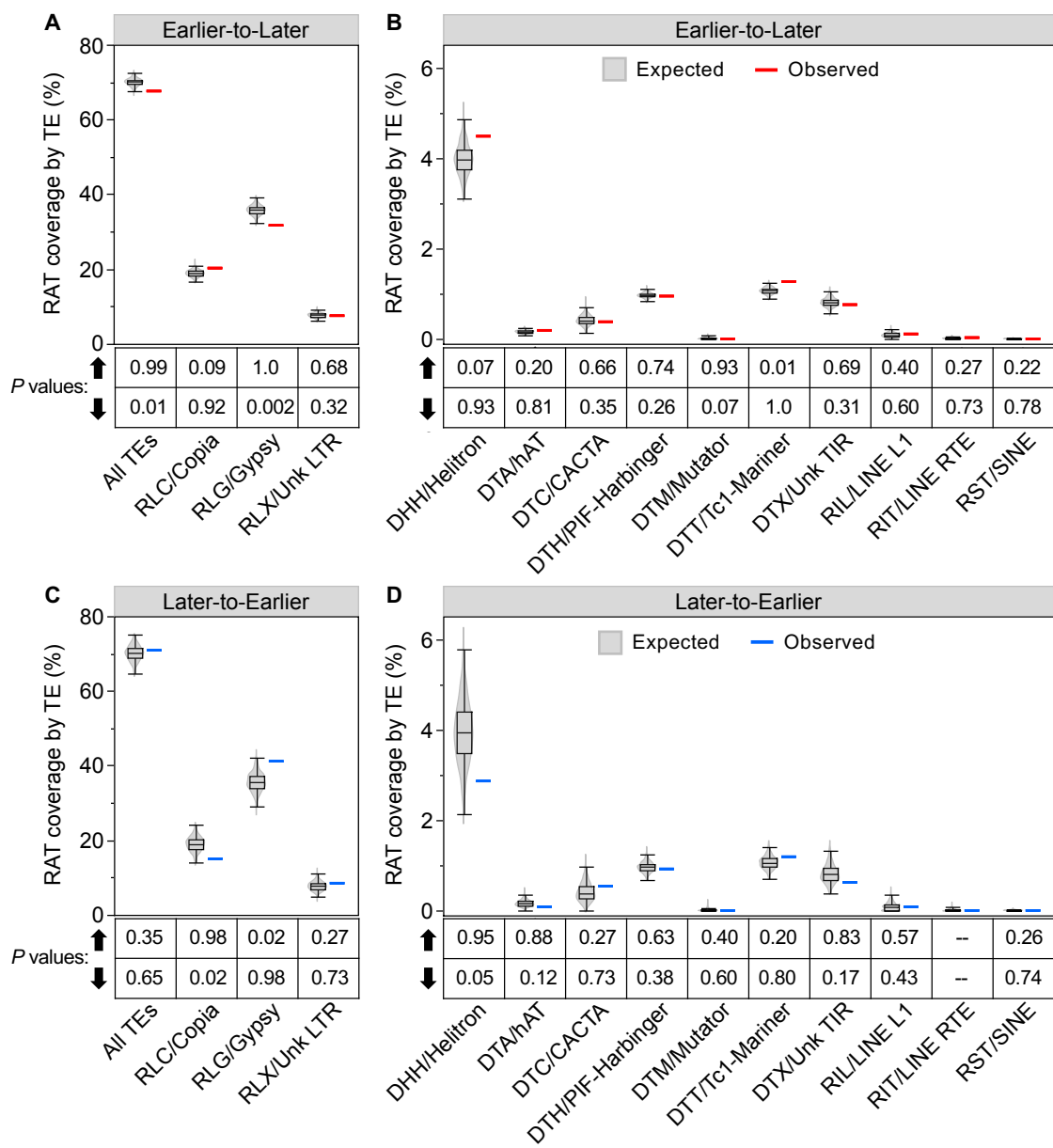

Supplement: S9 Fig — The percent coverage of all TEs and individual TE superfamilies annotated in the B73 RefGen_v4 genome was calculated for Earlier-to-Later (A and B) and Later-to-Earlier (C and D) non-CEN RATs and corresponding 1000 randomly shuffled sets (see Methods). The observed percentage for RATs (red or blue lines) are plotted alongside the expected frequency distribution of the random sets (grey violin plots overlaid with boxplots). Permutation P values below the graphs were calculated from the proportion of the 1000 random sets that have percent coverage values greater than (up arrow) or less than (down arrow) the observed value. No overlap was observed between Later-to-Earlier RATs and the RIT/LINE RTE superfamily, thus no P value was calculated. (PDF) [file pgen.1008623.s010.pdf]

S10 Fig.

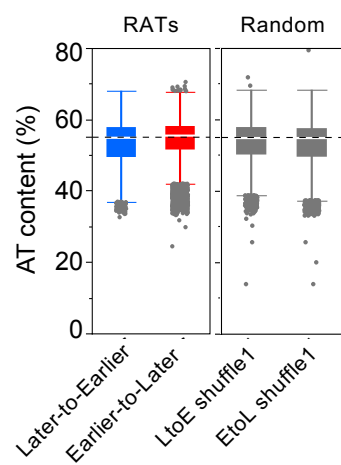

Supplement: S10 Fig — (A) The distributions of percent AT content, calculated in 3-kb static windows, for Later-to-Earlier and Earlier-to-Later non-CEN RATs and the corresponding random shuffle sets are plotted as boxplots. Values outside the boxplot whiskers (1.5 x IQR) are represented as grey dots. The dashed line indicates the genome wide median value. (PDF) [file pgen.1008623.s011.pdf]

S11 Fig.

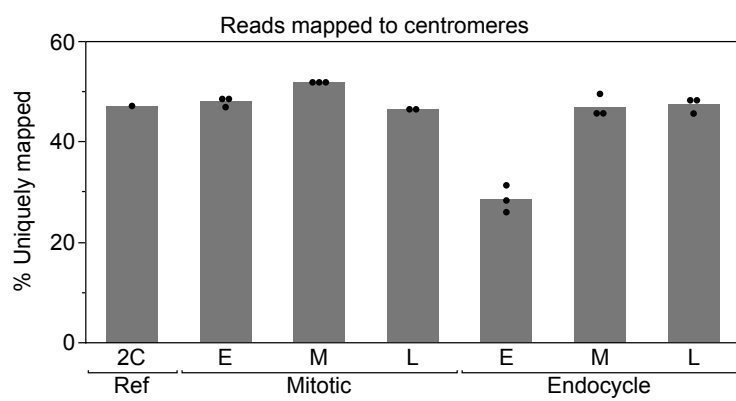

Supplement: S11 Fig — The average percentage of centromeric reads that map to unique locations is shown for each Repli-seq sample. Black dots represent the individual values for biological replicates. (PDF) [file pgen.1008623.s012.pdf]

S12 Fig.

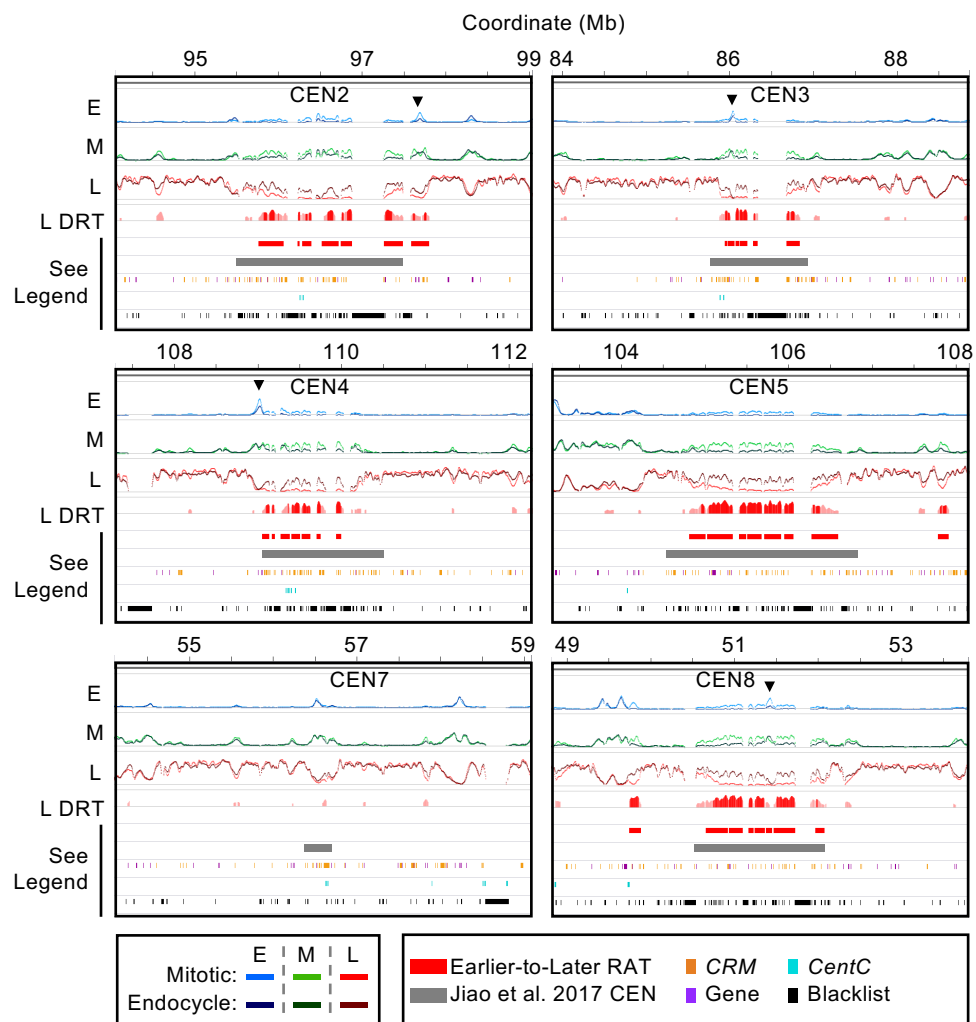

Supplement: S12 Fig — 5-Mb regions are shown for complex CENs 2, 3, 4, 5, and 8 and simple CEN 7. See main text Fig 4 legend for description. (PDF) [file pgen.1008623.s013.pdf]

S13 Fig.

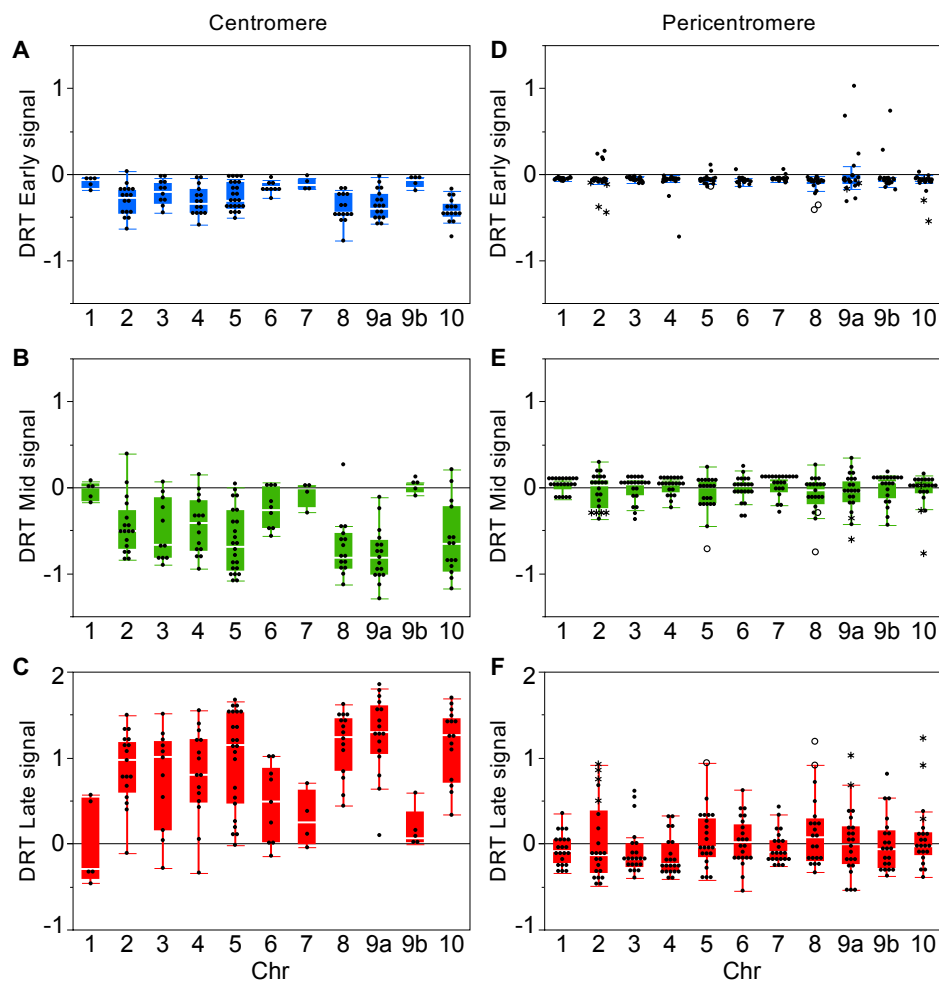

Supplement: S13 Fig — DRT values (endocycle minus mitotic) were calculated from early (A and D), mid (B and E) and late (C and F) RT profiles for each centromere and corresponding pericentromere (± 1 Mb) in 100-kb static windows. In panels D, E, and F asterisks indicate DRT values from windows where an Earlier-to-Later-CEN RAT extends past the called CEN boundary [46] into the pericentromere; open circles indicate windows that contain a non-CEN Earlier-to-Later RAT that met our compensation criteria. (PDF) [file pgen.1008623.s014.pdf]

S14 Fig.

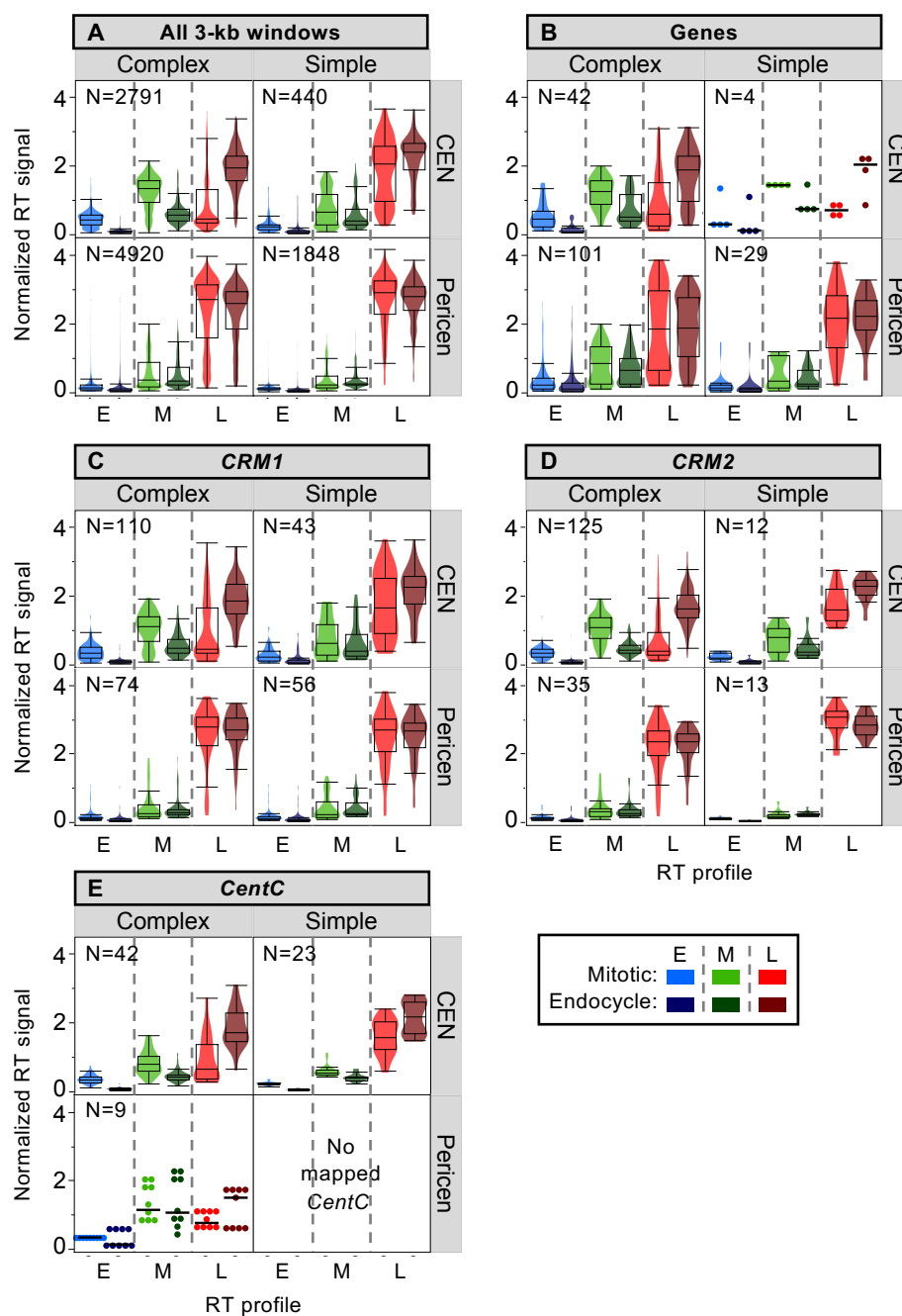

Supplement: S14 Fig — (A–E) The distributions of replication signals in early (E), mid (M), and late (L) during mitotic and endocycle S phases for all 3-kb windows (A), annotated genes (B), CRM1 elements (C), CRM2 elements (D), and mapped CentC repeats (E) in centromeres and pericentromeres (± 1 Mb). All elements within centromeres and pericentromeres are included, not just those that cover at least half of a 3-kb window, as in Fig 5. See main text Fig 5 legend for further description. (PDF) [file pgen.1008623.s015.pdf]

S15 Fig.

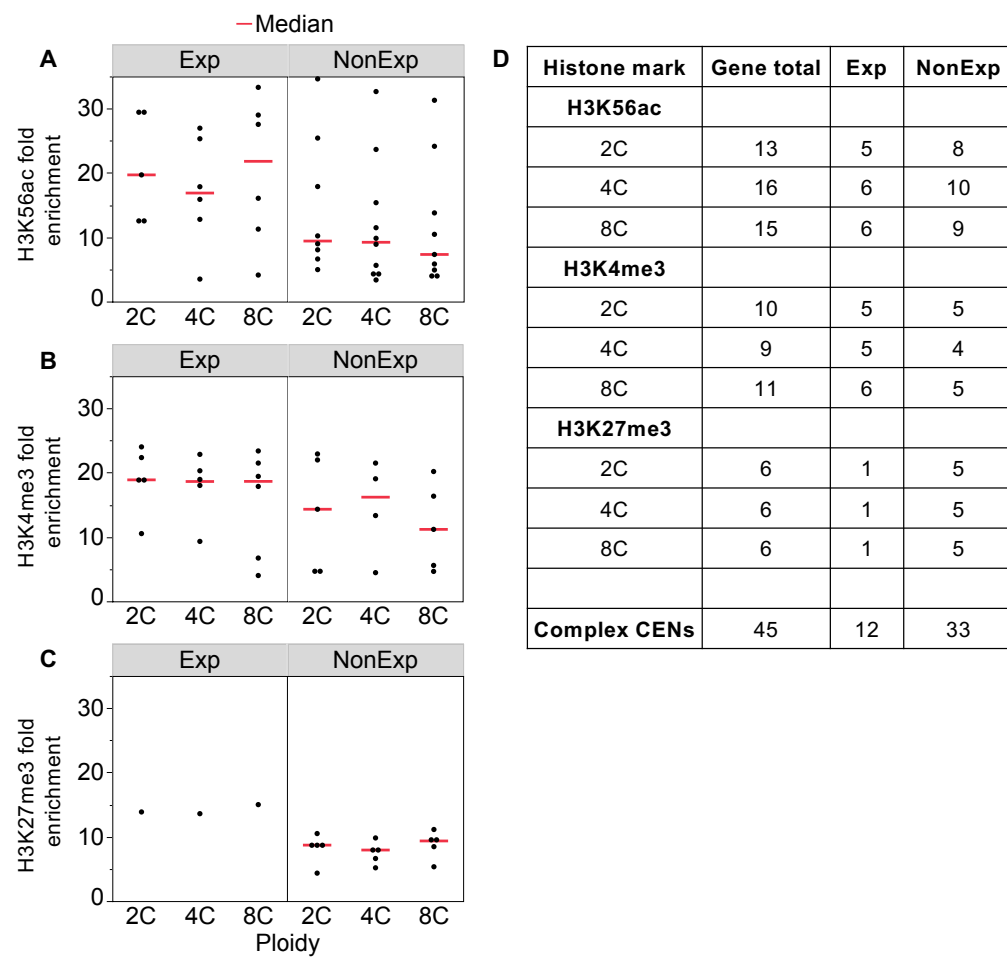

Supplement: S15 Fig — ChIP-seq data were generated for H3K56ac, H3K4me3 (active transcription) and H3K27me (repressive transcription) from 2C, 4C and 8C nuclei. (A–C) The fold enrichment values for peaks in expressed and non-expressed genes for H3K56ac (A), H3K4me3 (B) and H3K27me3 (C) in 2C, 4C and 8C nuclei. Red lines indicate the median value. (D) The number of expressed and non-expressed genes with each mark in 2C, 4C and 8C nuclei. (PDF) [file pgen.1008623.s016.pdf]

S16 Fig.

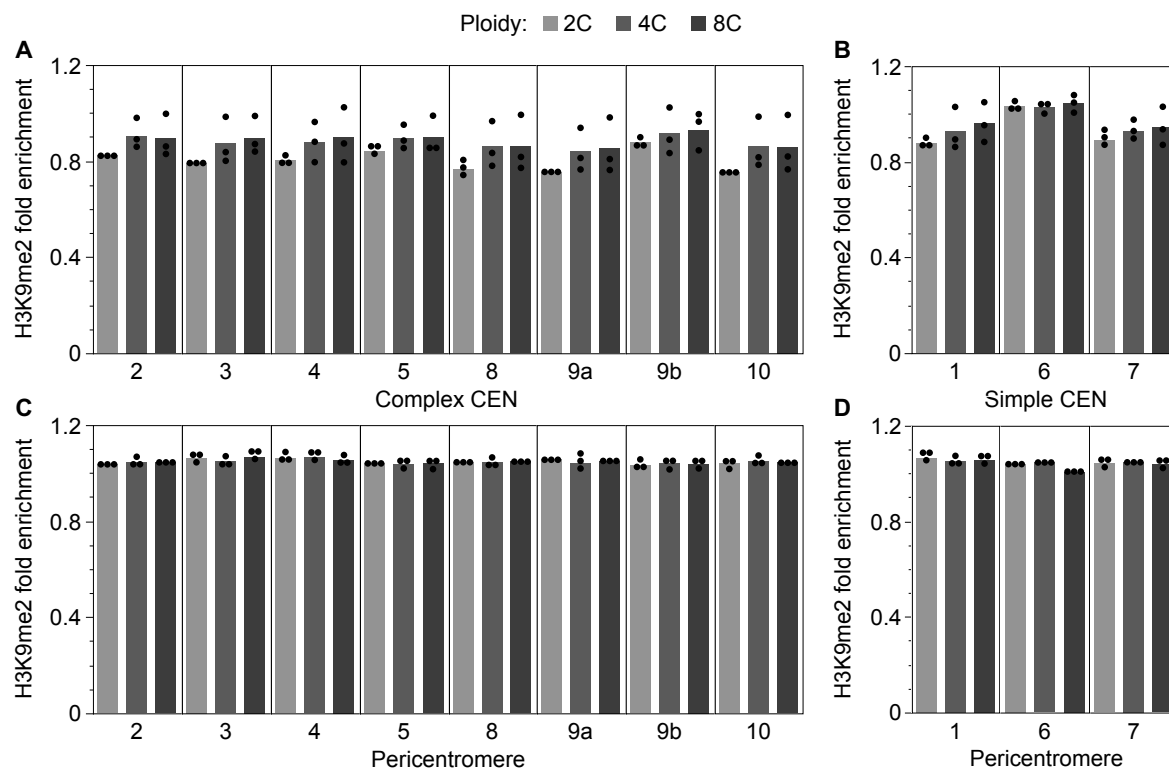

Supplement: S16 Fig — We used the ChIP-seq datasets from 2C, 4C and 8C nuclei to estimate the H3K9me2 average fold enrichment relative to DNA content by calculating the percent of total H3K9me2 reads found in a given centromere (A and B) using coordinates from [46] or pericentromere (C and D) and dividing by the percent of total input reads corresponding to that centromere or pericentromere. Black dots represent the individual values from biological replicates. (PDF) [file pgen.1008623.s017.pdf]

S17 Fig.

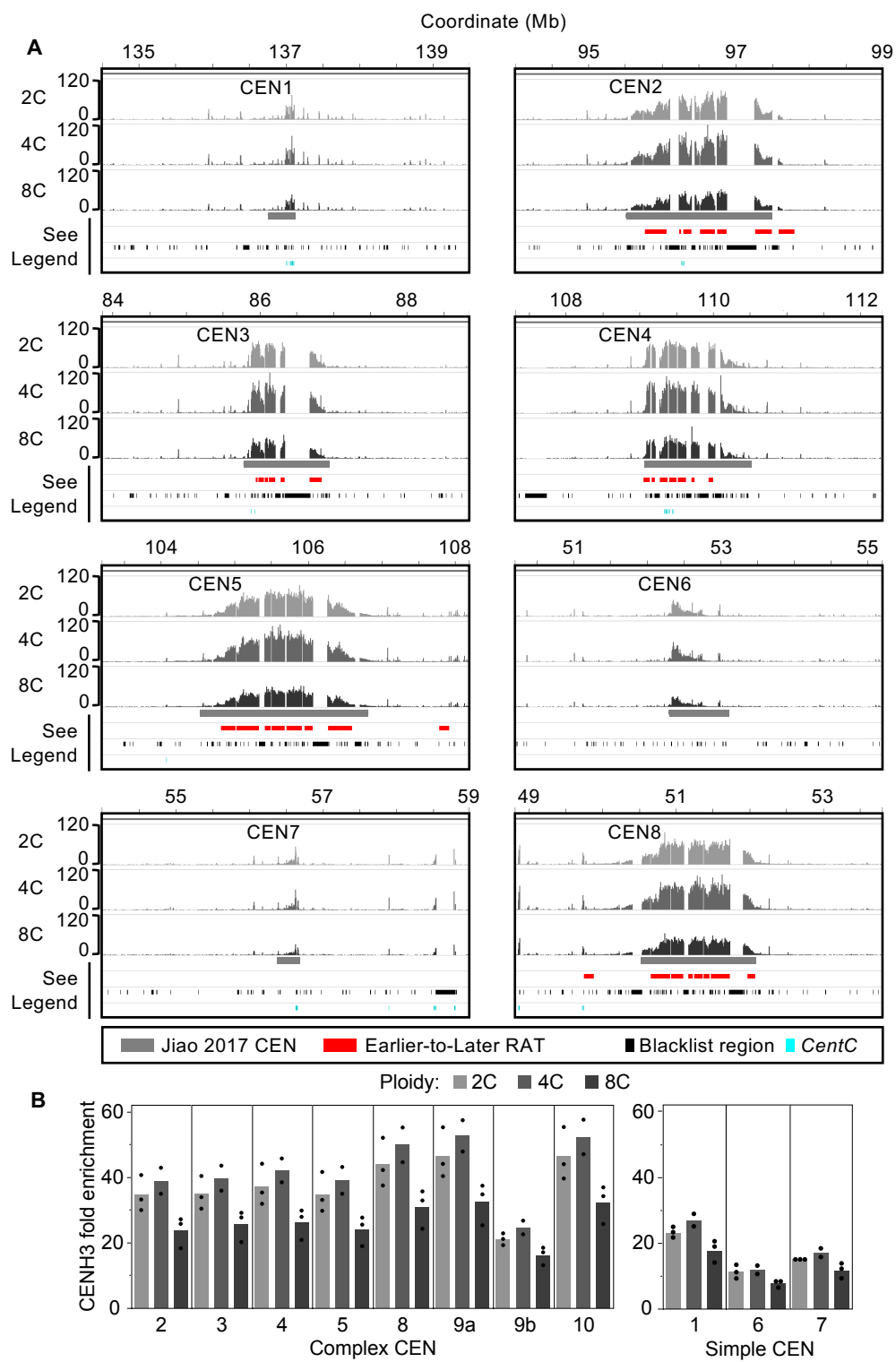

Supplement: S17 Fig — (A) CENH3 localization patterns for 2C, 4C and 8C nuclei for CEN 1–CEN 8. (B) CENH3 average fold enrichment relative to DNA content for complex and simple centromeres. See main text Fig 6 for CEN 9 and CEN 10 localization patterns and legend description. (PDF) [file pgen.1008623.s018.pdf]

S18 Fig.

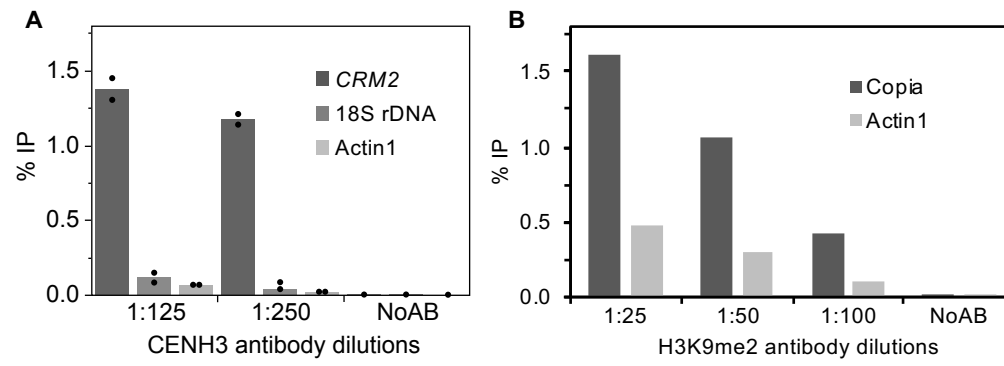

Supplement: S18 Fig — The percentage of input (% IP) was calculated for various antibody dilutions and primer sets for the Zea mays anti-CENH3 antibody (A) and anti-H3K9me2 antibody (B). Black dots in panel A represent the individual values from two biological replicates. Positive control primer sets (CRM2 and Copia retrotransposons) and negative control primer sets (18S rDNA and Actin1 UTR) were used. The no antibody control (NoAB) values are too small to see on the graph. See S1 Text for Supplemental Methods. (PDF) [file pgen.1008623.s019.pdf]
