# Supplementary material for: Comparing DNA replication programs reveals large timing shifts at centromeres of endocycling cells in maize roots
Source: PLoS Genet. 2020 Oct 14;16(10):e1008623. doi: 10.1371/journal.pgen.1008623 (PMC7588055; doi:10.1371/journal.pgen.1008623)
Supplement: S7 Fig — To assess whether changes in selected histone modifications related to gene transcription and chromatin accessibility occur in RATs, ChIP-seq data was generated for H3K56ac and H3K4me3 (active transcription and early replication) and H3K27me (repressive transcription and facultative heterochromatin) from sorted non S-phase 2C, 4C and 8C nuclei. (A–C) The distributions of fold enrichment values for H3K56ac (A), H3K4me3 (B) and H3K27me3 (C) peaks in expressed and non-expressed genes (see S1 Text) in 2C, 4C and 8C nuclei are plotted as boxplots for Later-to-Earlier and Earlier-to-Later RATs and their corresponding randomly shuffled sets (see Methods). Asterisks indicate statistically significant differences by the non-parametric Steel-Dwass-Critchlow-Fligner test at the following P value levels: ***, P < 0.0001; **, P < 0.001; *, P < 0.01. The increase in the fold enrichment of H3K56ac for expressed genes in Earlier-to-Later RATs (panel A) may be associated with increases in peak enrichment we observed near the 3' end of some genes. (D) The count and percentage of expressed and non-expressed genes with each histone modification shown in the boxplots in panels A–C. The 8C/2C ratio of genes with each mark is also shown to demonstrate there is very little change in the number of genes with each mark. The total number of expressed and non-expressed genes in each RAT or random category are shown at the bottom for reference. (PDF) [file pgen.1008623.s008.pdf]

S7 Fig.

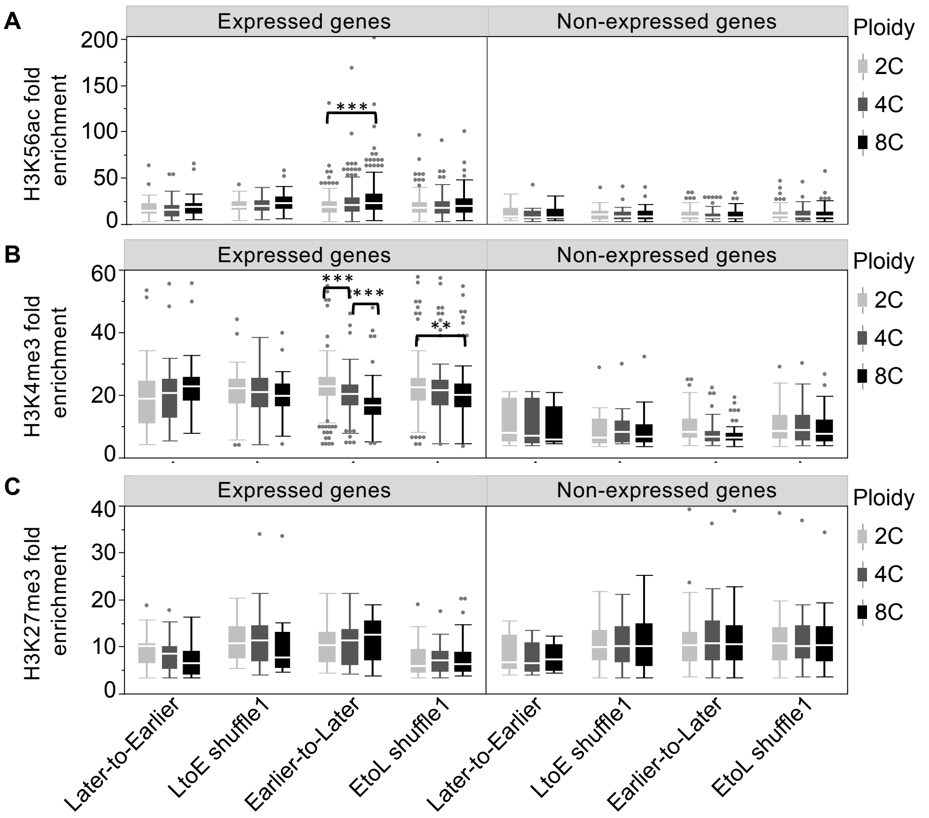

D

Histone mark	Later-to-Earlier genes		LtoE shuffle1 genes		Earlier-to-Later genes		EtoL shuffle1 genes	
	**Exp (%)**	**NonExp (%)**	**Exp (%)**	**NonExp (%)**	**Exp (%)**	**NonExp (%)**	**Exp (%)**	**NonExp (%)**
**H3K56ac**								
2C	46 (88.5)	14 (29.2)	59 (86.8)	29 (40.8)	256 (87.7)	78 (26.8)	243 (88.4)	79 (29.8)
4C	46 (88.5)	17 (35.4)	59 (86.8)	31 (43.7)	261 (89.4)	90 (30.9)	245 (89.1)	95 (35.8)
8C	46 (88.5)	17 (35.4)	58 (85.3)	29 (40.8)	256 (87.7)	77 (26.5)	239 (86.9)	88 (33.2)
8C/2C Ratio	1.00	1.21	0.98	1.00	1.00	0.99	0.98	1.11
**H3K4me3**								
2C	45 (86.5)	8 (16.7)	57 (83.8)	22 (31.0)	244 (83.6)	35 (12.0)	230 (83.6)	51 (19.2)
4C	46 (88.5)	7 (14.6)	58 (85.3)	14 (19.7)	242 (82.9)	34 (11.7)	231 (84.0)	44 (16.6)
8C	46 (88.5)	12 (25.0)	58 (85.3)	22 (31.0)	243 (83.2)	40 (13.7)	233 (84.7)	59 (22.3)
8C/2C Ratio	1.02	1.50	1.02	1.00	1.00	1.14	1.01	1.16
**H3K27me3**								
2C	21 (40.4)	14 (29.2)	13 (19.1)	28 (39.4)	20 (6.8)	82 (28.2)	35 (12.7)	87 (32.8)
4C	19 (36.5)	14 (29.2)	13 (19.1)	27 (38.0)	21 (7.2)	80 (27.5)	31 (11.3)	84 (31.7)
8C	17 (32.7)	14 (29.2)	13 (19.1)	28 (39.4)	22 (7.5)	86 (29.6)	33 (12.0)	89 (33.6)
8C/2C Ratio	0.81	1.00	1.00	1.00	1.10	1.05	0.94	1.02
**Total gene no.**	52	48	68	71	292	291	275	265
