# Supplementary material for: Comparing DNA replication programs reveals large timing shifts at centromeres of endocycling cells in maize roots
Source: PLoS Genet. 2020 Oct 14;16(10):e1008623. doi: 10.1371/journal.pgen.1008623 (PMC7588055; doi:10.1371/journal.pgen.1008623)
Supplement: S1 Table — The difference in replication signal between mitotic and endocycle profiles (endocycle minus mitotic) was calculated for each 3-kb window across the genome. The maximum negative difference value, which indicates a higher signal in the mitotic cycle, and the maximum positive difference value, which indicates a higher signal in the endocycle, are shown for early and late profiles. The average total difference range between these two values was used to calculate percentage thresholds for identifying RATs (see S2 Table and main text). (DOCX) [file pgen.1008623.s020.docx]

**S1 Table. Replication timing signal differences and thresholds.**

| **S-phase fraction** | **Average across chromosomes** | | | **10% threshold** | **25% threshold** |
| --- | --- | --- | --- | --- | --- |
|  | **Negative difference max** | **Positive difference max** | **Total difference range** |  |  |
| **Early** | -2.6 | 1.9 | 4.5 | -- | -- |
| **Late** | -1.6 | 1.9 | 3.5 | -- | -- |
| **Average** | -2.1 | 1.9 | 4.0 | 0.4 | 1.0 |
