# Supplementary material for: Comparing DNA replication programs reveals large timing shifts at centromeres of endocycling cells in maize roots
Source: PLoS Genet. 2020 Oct 14;16(10):e1008623. doi: 10.1371/journal.pgen.1008623 (PMC7588055; doi:10.1371/journal.pgen.1008623)
Supplement: S2 Table — The thresholds from S1 Table (≥ 10% or ≥ 25%) were used to identify regions with DRT in early or late S phase that were compensated by difference(s) with an opposite sign in one or both of the other two S-phase fractions (early + mid or mid + late) with greater than or equal to the same magnitude. The count, minimum, maximum and median region size, and the total coverage of the B73 RefGen_v4 genome are shown. Final robust RATs included at least one core region with a ≥ 25% DRT, but immediately adjacent regions of ≥ 10% DRT were merged together with the ≥ 25% regions to identify larger regions of contiguous change. (DOCX) [file pgen.1008623.s021.docx]

**S2 Table. Summary statistics of preliminary RAT calling steps.**

| **RT change** | **Stringency threshold** | **Region count** | **Min size (kb)** | **Median size (kb)** | **Max size (kb)** | **Sum (kb)** | **Genome coverage (%)** |
| --- | --- | --- | --- | --- | --- | --- | --- |
| **Earlier-to-Later** | ≥ 10% | 2626 | 3 | 39 | 1110 | 144,996 | 6.9 |
|  | ≥ 25% | 284 | 3 | 27 | 543 | 12,468 | 0.6 |
|  | **Final RATs** | **233** | **24** | **135** | **1110** | **34,575** | **1.6** |
| **Later-to-Earlier** | ≥ 10% | 2180 | 3 | 30 | 357 | 87,936 | 4.2 |
|  | ≥ 25% | 49 | 3 | 24 | 102 | 1,494 | 0.1 |
|  | **Final RATs** | **41** | **60** | **141** | **315** | **6,291** | **0.3** |
