# Supplementary material for: Comparing DNA replication programs reveals large timing shifts at centromeres of endocycling cells in maize roots
Source: PLoS Genet. 2020 Oct 14;16(10):e1008623. doi: 10.1371/journal.pgen.1008623 (PMC7588055; doi:10.1371/journal.pgen.1008623)
Supplement: S3 Table — The percent of RATs that contain genes, the total number of genes and expressed genes and the mean gene count per RAT are shown. (DOCX) [file pgen.1008623.s022.docx]

**S3 Table. Gene summary in non-centromeric RATs.**

|  | **Region count** | **Genome coverage (%)** | **RATs with gene (%)**^a^ | **RATs with expressed gene (%)**^a^ | **Gene count** | | |
| --- | --- | --- | --- | --- | --- | --- | --- |
|  |  |  |  |  | **Total** | **Expressed^b^**  **(% of total)** | **Mean No.**  **per region^c^** |
| **Later-to-Earlier** | 41 | 0.3 | 38 (92.7) | 34 (82.9) | 100 | 52 (52.0) | 2.6 |
| **Earlier-to-Later** | 192 | 1.3 | 185 (96.4) * | 175 (91.1) * | 582 | 292 (50.2) | 3.2 |
| ***Total*** | 233 | 1.6 | 223 (95.7) | 209 (89.7) | 682 | 344 (50.4) | 3.1 |
| Footnotes:  ^a^ Asterisks indicate values that were significantly greater than random expectation (P value = 0.001) from permutation analysis. See Fig 3.  ^b^ Expressed genes were defined as those having an FPKM ≥ 1 in at least one of two root specific RNA-seq  datasets (see S1 Text for details).  ^c^ The mean count of genes per region (including all genes). | | | | | | | |
