# Supplementary material for: Comparing DNA replication programs reveals large timing shifts at centromeres of endocycling cells in maize roots
Source: PLoS Genet. 2020 Oct 14;16(10):e1008623. doi: 10.1371/journal.pgen.1008623 (PMC7588055; doi:10.1371/journal.pgen.1008623)
Supplement: S4 Table — We calculated the total number of 3-kb windows in complex centromeres and pericentromeres (± 1 Mb), as well as the number of windows that show DRT values that are compensated (threshold ≥ 10%) by equal and opposite shifts in the other two S-phase fractions. (DOCX) [file pgen.1008623.s023.docx]

**S4 Table. Compensated differences in RT in complex centromeres and corresponding pericentromeres.**

|  | **Total 3-kb**  **window count** | **Earlier-to-Later shift** | | **Later-to-Earlier shift** | |
| --- | --- | --- | --- | --- | --- |
|  |  | **Windows compensated**  **at** ≥ **10%** | **% of total** | **Windows compensated**  **at** ≥ **10%** | **% of total** |
| **Centromeres** | 2,791 | 2,384 | 85.4 | 0 | 0.0 |
| **Pericentromeres** | 4,757^a^ | 372 | 7.8 | 77 | 1.6 |
| Footnotes:  ^a^ Windows that were identified as presumed CEN RAT (Table 2) and were outside the published centromere boundary were excluded from the pericentromere counts. | | | | | |
