# Supplementary material for: Comparing DNA replication programs reveals large timing shifts at centromeres of endocycling cells in maize roots
Source: PLoS Genet. 2020 Oct 14;16(10):e1008623. doi: 10.1371/journal.pgen.1008623 (PMC7588055; doi:10.1371/journal.pgen.1008623)
Supplement: S5 Table — CENH3 fold enrichment relative to DNA content and the ratio of enrichments between 4C and 2C and 8C and 2C are shown for each centromere. Fold enrichment values are the mean ± S. D. of three biological replicates for 2C and 8C and two biological replicates of 4C. See main text Fig 6 legend for further description. Two sets of theoretical ratio values are also presented. The first set, labeled “proportional redeposition”, corresponds to the hypothesis that CENH3 is diluted relative to total DNA during replication, and is then redeposited to a level proportional to the DNA content during the subsequent gap phase. The second set, labeled “no redeposition”, corresponds to an alternate hypothesis that CENH3 is diluted relative to total DNA during replication, and is not redeposited in the subsequent gap phase. (DOCX) [file pgen.1008623.s024.docx]

**S5 Table. CENH3 average fold enrichment relative to DNA content in centromeres.**

| **CEN** | **CENH3 average fold enrichment (± S. D.)** | | | **Ratio values** | |
| --- | --- | --- | --- | --- | --- |
|  | **2C** | **4C** | **8C** | **4C/2C** | **8C/2C** |
| 1 | 23.1 ± 1.5 | 27.0 ± 2.7 | 17.7 ± 3.2 | 1.17 | 0.77 |
| 2 | 36.2 ± 5.8 | 40.7 ± 5.9 | 24.6 ± 4.8 | 1.11 | 0.68 |
| 3 | 36.2 ± 5.3 | 41.2 ± 5.7 | 26.5 ± 4.9 | 1.13 | 0.73 |
| 4 | 39.5 ± 6.7 | 44.8 ± 5.5 | 27.9 ± 5.0 | 1.12 | 0.71 |
| 5 | 35.6 ± 6.3 | 39.9 ± 5.9 | 24.6 ± 4.6 | 1.11 | 0.69 |
| 6 | 11.4 ± 2.1 | 12.0 ± 1.9 | 7.8 ± 1.1 | 1.06 | 0.68 |
| 7 | 15.2 ± 0.6 | 17.3 ± 1.9 | 11.7 ± 2.1 | 1.13 | 0.77 |
| 8 | 45.1 ± 7.7 | 51.2 ± 7.7 | 31.7 ± 6.1 | 1.13 | 0.70 |
| 9a | 46.9 ± 7.8 | 53.0 ± 6.8 | 32.7 ± 6.4 | 1.11 | 0.70 |
| 9b | 21.3 ± 1.9 | 24.9 ± 3.0 | 16.3 ± 2.7 | 1.17 | 0.76 |
| 10 | 46.7 ± 8.2 | 52.8 ± 7.5 | 32.5 ± 5.9 | 1.11 | 0.70 |
| **Theoretical values:** | | | | | |
| **Proportional redeposition** | | | | 1.0 | 1.0 |
| **No redeposition** | | | | 0.5 | 0.5 |
